# Simple Bone Cyst Within Florid Cemento-Osseous Dysplasia: A Report of Two Cases

**DOI:** 10.7759/cureus.65803

**Published:** 2024-07-30

**Authors:** Marie Rollin, Ihsene Taihi

**Affiliations:** 1 Oral Surgery, Hospital Saint-Antoine, Paris, FRA; 2 Odontology, Université Paris Cité, Paris, FRA; 3 Oral Surgery, Hôpital Rothschild, Paris, FRA; 4 URP 2496 Laboratory of Orofacial Pathologies, Imaging, and Biotherapies, Université Paris Cité, Montrouge, FRA

**Keywords:** dentistry, oral surgery, oral medicine, oral pathology, simple bone cyst, florid cemento-osseous dysplasia

## Abstract

Florid cemento-osseous dysplasia (FCOD) can rarely be associated with bone lesions, including simple bone cysts (SBCs). Only a few cases showing the co-occurrence of these two distinct entities have been reported in the literature. This article reports two new cases of SBCs within FCOD. The first case involves a 37-year-old Black female with a large radiolucent lesion around the apex of the right third mandibular molar, accompanied by multiple cemento-osseous lesions around the mandibular teeth. Surgical exploration revealed an empty bone cavity, confirming the diagnosis of an SBC. Curettage of the bone walls was performed to stimulate healing, with promising results observed at the nine-month follow-up. The second case concerns a 44-year-old Black female presenting with a radiolucent lesion at the site of extraction of the left third mandibular molar and a slightly painful radiolucent/radio-opaque lesion in the apical region of the right first mandibular molar. Surgical exploration confirmed an SBC in the region of the left third mandibular molar and a bone biopsy was made. Histopathological analysis confirmed FCOD. Curettage of the bone wall was again used to promote healing through increased bleeding. At the 30-month follow-up, new dysplastic lesions had appeared, the initial SBC had healed completely, and a new SBC seemed to have developed in the apical region of the left second mandibular premolar. These cases highlight the importance of considering SBCs in the differential diagnosis of well-defined radiolucent lesions and demonstrate that surgical intervention for SBC-associated FCOD can yield favorable outcomes. From these cases, we learn the critical need for accurate diagnosis to avoid unnecessary treatments and the value of regular follow-up to monitor for recurrence or new lesions.

## Introduction

Cemento-osseous dysplasia (COD) presents in three forms: focal, periapical, and florid. The florid type was first described by Melrose in 1976 [1]. COD lesions progress through different stages: osteoporotic, cementoblastic, and mature [2].

Florid COD (FCOD) predominantly affects middle-aged and elderly Black women, with lesions primarily occurring in the mandible. As a benign odontogenic lesion of the jaws, FCOD is typically managed through bi-annual to annual monitoring without the need for surgical intervention [2].

FCOD has been reported in association with various other osseous lesions including aneurysmal cysts, central giant cell granuloma, and glandular odontogenic cysts [3-5]. In 1976, Melrose et al. first described a case of a simple bone cyst (SBC) within FCOD [1]. SBCs, often referred to as traumatic bone cysts, are non-epithelial formations surrounded by bone walls, containing either no material or liquid and/or connective tissue [6]. The World Health Organization (WHO) retains the term “simple bone cyst” for this condition [2].

The co-occurrence of FCOD with other osseous lesions may be underdiagnosed due to similar radiographic appearances and the lack of systematic surgical exploration. Slightly painful lesions might lead to unnecessary root canal treatments or tooth extractions, which could increase the risk of new dysplastic lesions in these areas. Radiographic monitoring is essential to detect any associated osseous lesions that may require treatment.

In this article, we present two patients diagnosed with both SBC and FCOD lesions, discuss their management, and explore the characteristics and potential mechanisms underlying this rare association and the importance of early diagnosis and follow-up.

## Case presentation

Case 1

A 37-year-old Black female was referred by her dentist for a large, painless radiolucent lesion located in the apical region of the right third mandibular molar. Oral examination revealed no facial or intraoral swelling and no lymphadenopathies. The lower right third molar was partially erupted and mesially inclined, while the lower right second molar was absent. The pulp vitality test for the lower right third molar was positive. The patient reported no discomfort, infectious episodes, or loss of sensitivity in the inferior alveolar nerve region.

**Figure 1:**
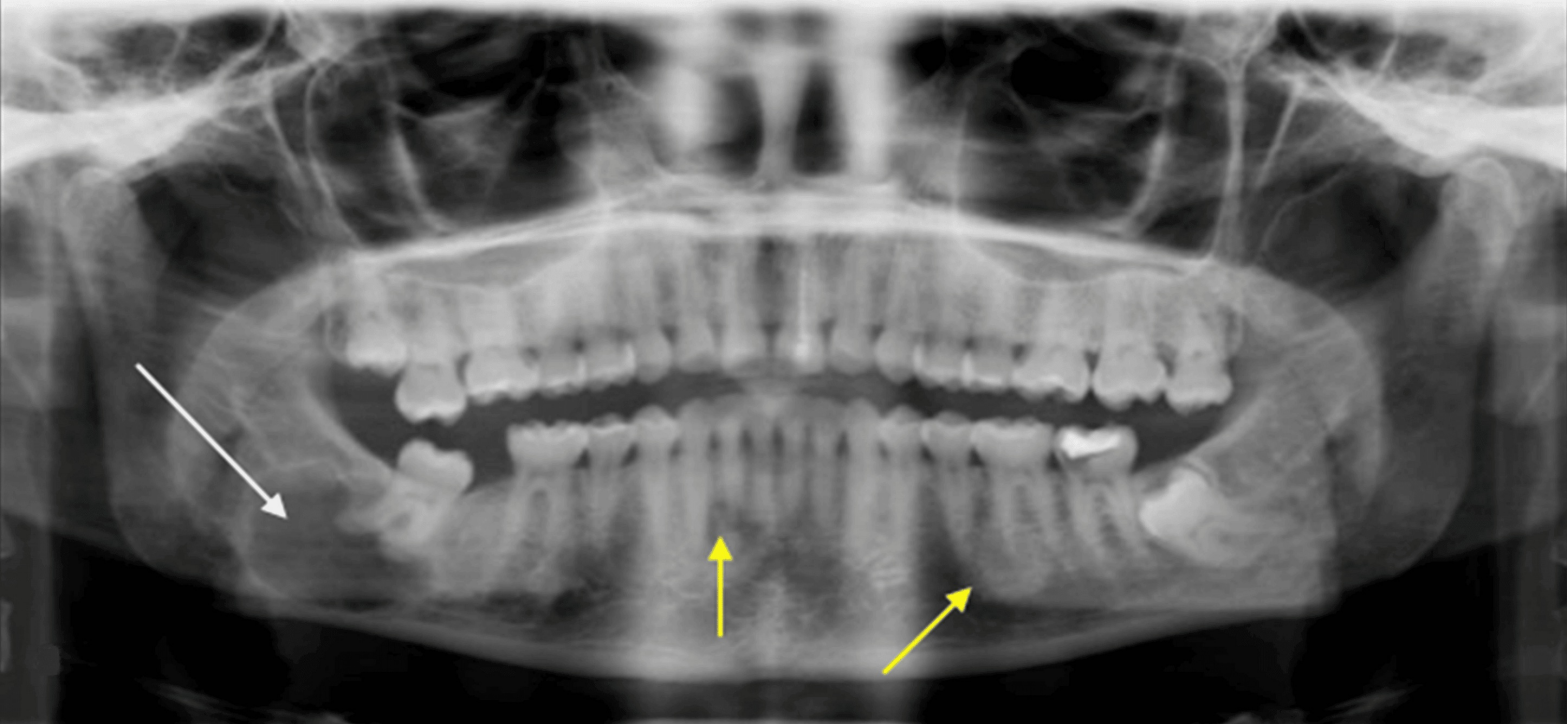
Case 1: Initial panoramic radiograph showing a large radiolucent lesion below the lower right third molar roots (white arrow) and multiple FCOD lesions around the apex in the mandibular premolars and molars regions (yellow arrows) FCOD: florid cemento-osseous dysplasia

**Figure 2:**
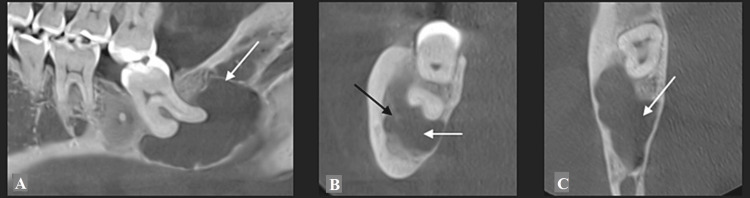
Case 1: (A) CT scan showing the radiolucent lesion in the apical region of the lower right third molar (white arrow); (B) The inferior alveolar nerve is partially included in the lesion and pushed back into the buccal side (black arrow); (C) A slight buccal expansion of the lesion with a thin cortical buccal bone.

Panoramic radiography (Figure 1) showed a large radiolucent lesion in the apical region of the lower right third molar, homogenous with a well-defined radiopaque border, invading the inferior alveolar nerve region. Multiple heterogeneous FCOD lesions were simultaneously noted, ill-defined around the apex of several mandibular teeth. Computed tomography (CT) revealed a large radiolucent lesion measuring 2x2.5 cm (Figure 2A). The inferior alveolar nerve was buccal or partially included in the lesion (Figure 2B). A slight buccal bone expansion was observed on the sagittal section (Figure 2C). The lesion's limits were well-defined, and the lingual cortical bone was thin.

Differential diagnoses included an odontogenic cyst of inflammatory origin associated with the lower right third molar, a dentigerous cyst, an odontogenic keratocyst, a unicystic ameloblastoma, or an SBC due to the radiographic appearance of a radiolucent lesion surrounding the apical region of the lower right third molar.

**Figure 3:**
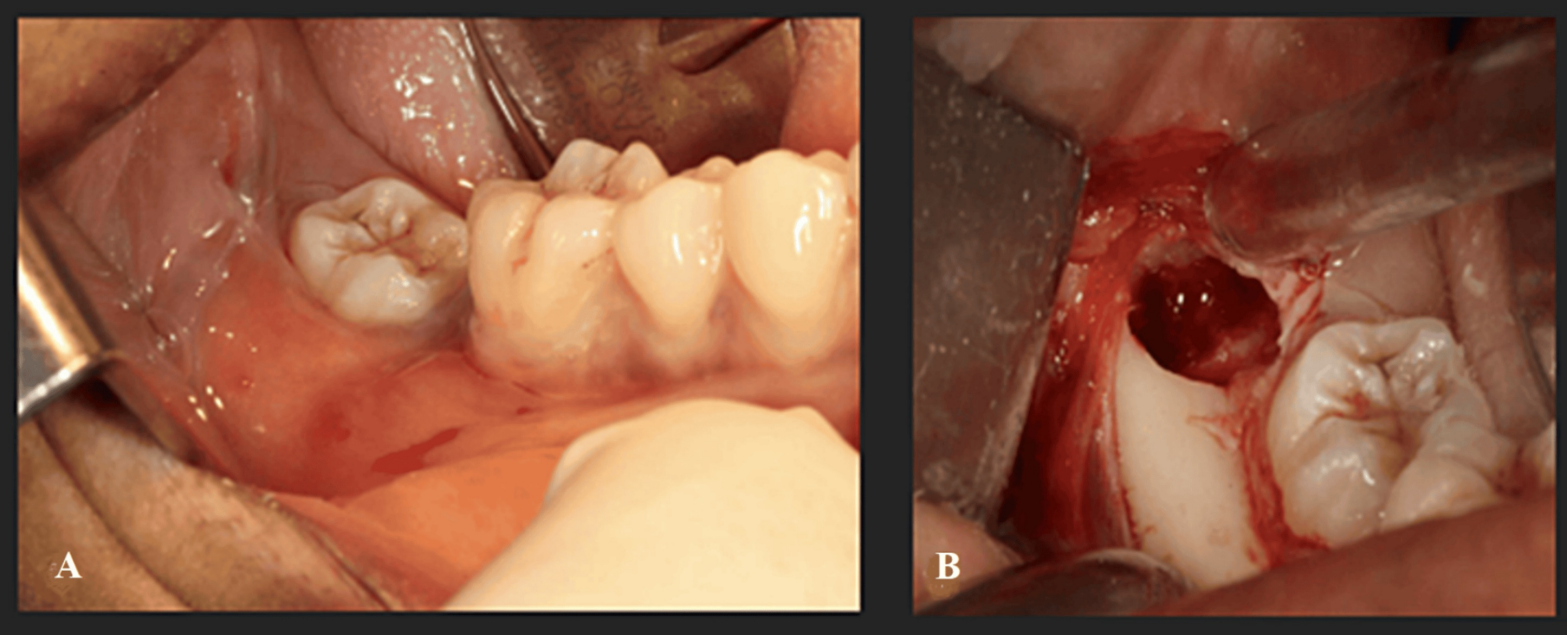
Case 1: (A) Intra-oral views of the mandibular right molar region showing the infra-position of the lower right third molar; (B) Surgical exploration: an empty cystic cavity is visible behind the lower right third molar, confirming the diagnosis of a simple bone cyst

Surgical exploration of the cyst was planned. During the intervention, after elevating the flap and creating a small bone window, the cystic cavity was found to be empty (Figure 3), excluding epithelial cysts from the differential diagnosis. The tooth was not extracted as it was asymptomatic, to avoid the risk of mandibular fracture. A bone fragment was sampled for histopathological analysis.

Histological findings showed bone-like tissue remodeled with fibrous tissue, with no signs of inflammation or malignancy. The bone tissue exhibited lamellar spans with osteocytes, and the inter-lamellar tissue contained non-inflammatory adipose and fibrous tissue. The final diagnosis for the radiolucent lesion was a SBC, and the histological analysis confirmed the diagnosis of FCOD affecting the bone surrounding the SBC.

**Figure 4:**
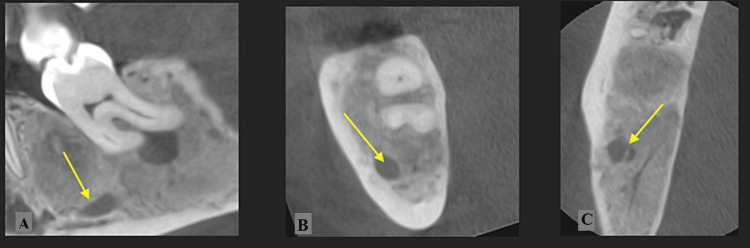
Case 1: (A) CT scan after nine months showing almost complete healing around the lower right third molar; (B, C) The inferior alveolar bone (yellow arrows) is well individualized.

After a nine-month follow-up, a new CT scan (Figure 4) showed a reduction in the lesion size around the lower right third molar, with almost complete bone formation. The inferior alveolar nerve was individualized, with bone formation all around. It was decided to extract the lower right third molar as the lesion did not heal completely. Since the risk of mandibular fracture was decreased and the tooth was non-functional, extraction was deemed necessary.

Case 2

A 44-year-old Black female was referred by her dentist after incidental discovery of multiple mandibular radiodense lesions on panoramic radiography. The patient reported slight jaw pain when chewing in the posterior mandibular regions for the past two years. Her medical history included systemic erythematous lupus treated with Plaquenil. Extra-oral examination was normal.

Intra-oral examination revealed no dental mobility and a slight, indurated, painless swelling on the buccal side of the right mandibular region. The initial radiographic examination showed a large radiolucent lesion in the left mandibular angle at the extraction site of the left third molar, with blurry outlines. Additional radiolucent/radiopaque mandibular lesions were observed in the apical regions of the lower premolars and molars. The CT scan revealed thinned and swollen buccal cortical bone.

A biopsy was performed on both the left mandibular lesion at the extraction site of the lower left third molar and the apical region of the lower right first molar. During surgical exploration, empty bone cavities were found at both sites, and curettage of the bone walls was performed to enhance healing. Histopathological findings confirmed the diagnosis of FCOD in the apical region of the lower right first molar and SBC at the extraction site of the lower left third molar.

**Figure 5:**
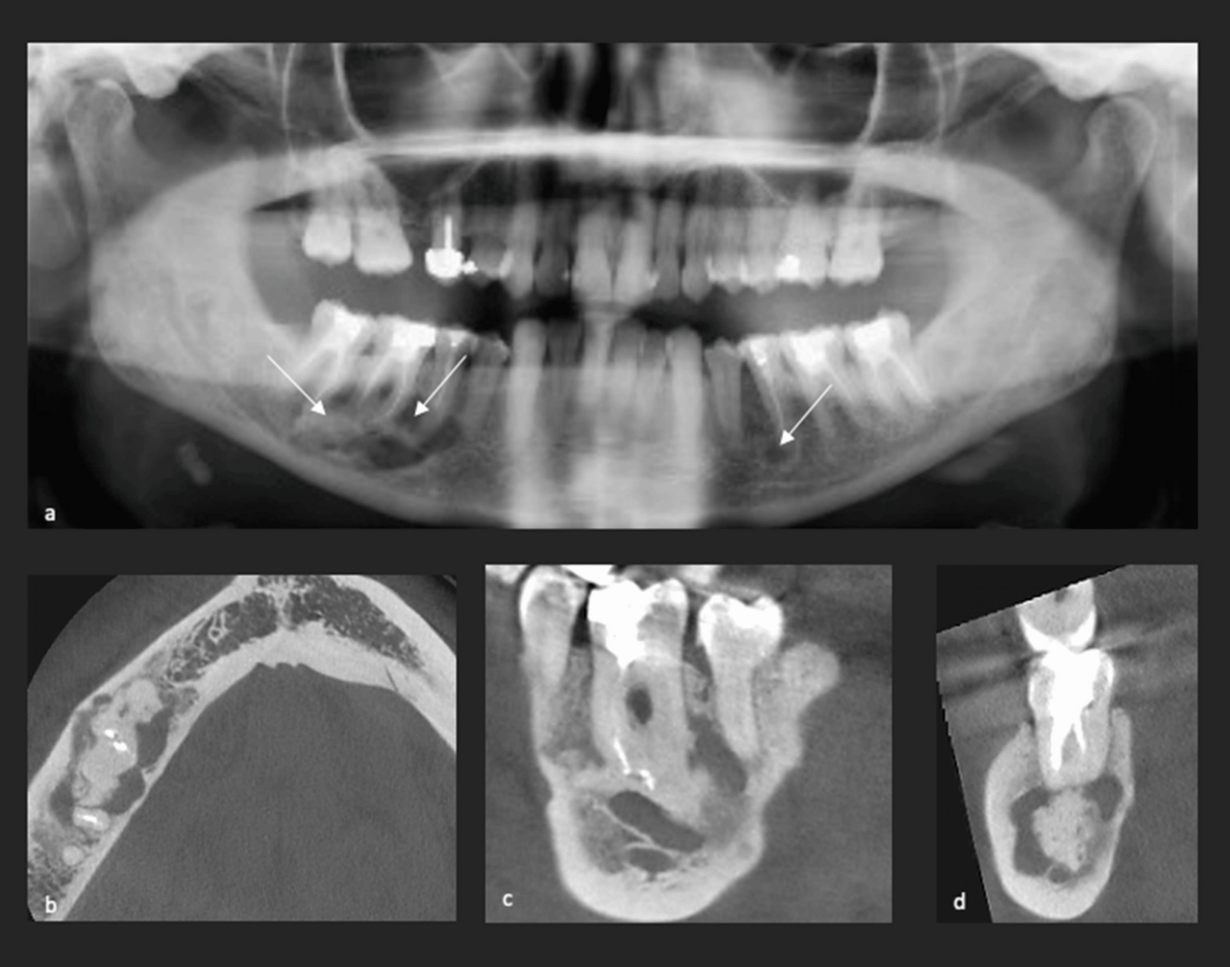
Case 2 (a) Panoramic radiograph after four months of bone curettage and biopsy showing complete healing of SBC in the extraction site of the lower left third molar (yellow arrow) and partial bone healing in the apical region of the lower right premolars and molars within the diagnosed of FCOD (white arrows); (b,c,d) CT scan at the two-year follow-up showing radiolucent/radiopaque mandibular FCOD lesions in the apical regions of the lower premolars and molars. It shows a partial healing of the lesion. SBC: simple bone cyst; FCOD: florid cemento-osseous dysplasia

At four months follow-up, panoramic radiography (Figure 5a) showed complete bone healing of the lesion at the extraction site of the lower left third molar and partial bone healing in the right mandibular molar region. The patient reported no pain, and no inflammation was noted on intra-oral examination.

The patient was followed for two years, during which she reported regression of previous chewing symptoms. Intra-oral examination revealed new, slightly painful vestibular palpation in the left mandibular region and slightly painful percussion on the lower left second premolar.

CT (Figure 5b, 5c, 5d) was performed to assess the evolution of the FCOD lesions and revealed slight expansion of cemento-osseous dysplastic lesions around the lower left second premolar and in the right posterior mandibular region. A control CT was performed two and a half years after the first visit due to persistent symptoms. It showed an enlargement of the radiolucent lesion around the apical region of the lower left second premolar with poorly defined outlines.

## Discussion

Due to the presence of both bone and cementum in dysplastic lesions, Waldron in 1985 [7] proposed changing the term “florid osseous dysplasia,” originally used by Melrose et al. [1], to “florid cemento-osseous dysplasia”. Waldron described these lesions as bilateral, symmetrical, and densely sclerotic, predominantly found in the mandibular molar or premolar regions. The association between FCOD and SBC was first reported in 1976 [1], with 14 SBCs observed among 34 patients exhibiting FCOD lesions.

The incidence of SBC in FCOD patients may be underreported. The empty bone cavities formed by FCOD could imply a systematic association between the two entities, but this has not been proven, as FCOD lesions are not consistently explored surgically. Conversely, SBCs might be overdiagnosed due to the similar radiographic appearance of FCOD lesions (radiolucent or mixed) and SBCs when no surgical exploration confirms the diagnosis. Melrose et al. [1] found SBCs in nearly half of the patients with FCOD lesions (14 of 34), but recent case reports show fewer associations, ranging from one [8-10] to eight [11] cases. In a 2021 study by Gumru et al., the prevalence of FCOD associated with SBC lesions was 5% (eight of 142 patients), indicating that the actual prevalence of SBCs in FCOD patients remains unclear [11].

The formation of SBC in FCOD patients could be explained by liquefaction necrosis or resorption of a blood clot following intramedullary hemorrhage in the FCOD lesion formation mechanism. This results in the destruction of the bone by enzymatic activity, leading to an empty bone cavity [12]. In Case 2, this process might explain the formation of the SBC following the trauma from the extraction of the lower left third molar, which was favored by the dysplastic bone. SBCs are usually painless, but as shown in our second case, they can be associated with swelling and slight pain upon palpation. SBCs predominantly affect the mandible. The primary ossification site of the mandible, near the mental foramen, could be the origin of most SBCs due to abnormalities in cellular differentiation during ossification [13]. Although rare, SBCs can also occur in the maxilla, with two cases reported since 1976 [1,14].

In these two cases, surgical exploration of the SBC with curettage of the bone walls appeared to aid in the healing process of the bone lesion. Stimulation of bone bleeding promotes bone formation. In cases of incidental discovery and asymptomatic SBCs, surgical exploration can be debated as it may potentially cause infection and/or exacerbate nearby FCOD lesions. Patients should have follow-up visits to monitor the healing and evolution of the lesions, as new SBCs can develop over time. Not surgically treating an SBC could increase the risk of mandibular fracture, as demonstrated in Case 1. There is a higher prevalence of recurrences when SBCs are associated with FCOD lesions (75%) [15].

Furthermore, FCOD can be associated with other bone lesions such as aneurysmal cysts, as shown by Yeom and Yoon [3] in a case report and literature review, as well as glandular odontogenic cysts, with one case reported by Kungoane and Robinson [4]. In 2017, Sarmento et al. reported a case of FCOD associated with a peripheral giant cell granuloma [5]. These associations, although rare in the literature, can be easily missed clinically and misdiagnosed as part of FCOD lesions. Lesions such as SBCs and aneurysmal cysts are the most common lesions associated with FCOD [3]. This could be due to the bone resorption and bone-cementum-like tissue formation characteristic of FCOD, which may facilitate the development of other lesions arising from these tissues.

## Conclusions

The diagnosis of the association between SBC and FCOD is very challenging due to their similar radiographic appearances and the lack of routine surgical exploration in such cases. These two reported cases confirm that this association may be underdiagnosed and highlight the importance of considering SBC as a differential diagnosis for well-defined radiolucent lesions. Surgical intervention for symptomatic SBCs demonstrated favorable outcomes in the presented cases, emphasizing the need for accurate diagnosis to avoid unnecessary treatments such as root canal therapy or tooth extraction. Regular follow-up is essential to monitor for recurrence or the emergence of new lesions, ensuring comprehensive management of these patients. Further studies are necessary to determine the true prevalence of SBCs within FCOD lesions and to develop standardized diagnostic and treatment protocols.
